# Adapted Training to Boost Upper Body Sensorimotor Control and Daily Living Functionality in Visually Impaired Baseball Players

**DOI:** 10.3390/medicina60071136

**Published:** 2024-07-15

**Authors:** Giuditta Carretti, Francesca Spano, Eleonora Sgambati, Mirko Manetti, Mirca Marini

**Affiliations:** 1 Department of Experimental and Clinical Medicine, Section of Anatomy and Histology, University of Florence, 50134 Florence, Italy; giuditta.carretti@unifi.it (G.C.); francesca.spano@edu.unifi.it (F.S.); mirko.manetti@unifi.it (M.M.); 2 Department of Biosciences and Territory, University of Molise, 86090 Pesche, Italy; eleonora.sgambati@unimol.it

**Keywords:** visual disability, adapted sport, blind baseball, sensorimotor training, daily living autonomy, proprioception, segmental coordination

## Abstract

*Background and Objectives:* Vision significantly contributes to postural control, balance, coordination, and body kinematics, thus deeply influencing everyday functionality. Sight-impaired subjects often show upper body anatomofunctional and kinetic chain alterations negatively impacting daily living efficiency and autonomy. The present study aimed to investigate and train, for the first time, upper body sensorimotor control in an Italian blind baseball team to boost global and segmental functionality while contemporarily prevent injuries. *Materials and Methods:* The whole team underwent a validated test battery using both quantitative traditional tools, such as goniometric active range of motion and muscular/functional tests, and an innovative biofeedback-based device, a Libra proprioceptive board. Consequently, a 6-week adapted training protocol was designed and leaded to improve sensorimotor control and, hence, counteract disability-related deficits and sport-specific overuse syndromes. *Results:* Statistically significant improvements were observed in all the investigated parameters. Noteworthy, an overall boost of global and segmental stability was detected through an orthostatic dynamic balance enhancement during the Y Balance test (*p* = 0.01) and trunk multiplanar control improvement on the Libra board (*p* = 0.01). Concurrently, the comparison of baseline vs. post-intervention outcomes revealed a consistent increase in upper body mobility (*p* < 0.05 for all the assessed districts), core recruitment (*p* = 0.01 for all the administered functional tests), and proprioceptive postural control (*p* = 0.01 for the Libra board validated test). *Conclusions:* Our findings suggest that a tailored sensorimotor training, conceived and led by an adapted physical activity kinesiologist, may effectively improve upper body functional prerequisites and global proprioceptive control, thus potentially promoting autonomy, quality of life, and physical activity/sport practice adherence in visually impaired individuals.

## 1. Introduction

Postural control requires a complex sensorimotor and cognitive input integration aimed to maintain balance and properly interact with the surrounding environment in response to external multimodal stimuli and perturbations [1,2,3]. Among these perceptual channels, vision plays a pivotal role in postural anchorage, spatial orientation, and movement accuracy, thus deeply conditioning daily living self-efficacy and autonomy [4,5]. Spatial representation through the selection and integration of multisensory signals is crucial to coherently perceive reality and interact with other individuals [6,7,8]. 

Vision, contemporarily providing spatial details and general background, deeply influences multisensory integration development and motivation/curiosity to explore the space-time dimension through movement during childhood [9,10,11,12]. In this regard, it has been demonstrated that the efficiency of multimodal interaction amongst different senses is mostly affected by perceptual and motor experiences during the aforementioned growth phase [13,14,15]. 

In case of early sight absence or loss, sensorimotor development delays and postural alterations may occur with a negative impact on stability, coordination, navigation, and socio-emotional wellbeing [9,16,17,18]. In this frame, even basic locomotor patterns become challenging tasks, hence increasing fall risk [19] and progressively discouraging self-engagement in recreational/sport activities, which triggers a vicious cycle toward sedentary habits able to jeopardize quality of life [20]. In order to perform daily living activities and sport practice safely and autonomously, visually impaired individuals count on vicariant senses, especially on the auditory and vestibular apparatus [21,22,23]. Both these sensory channels are anatomically located in the head, and therefore, head mobility and segmental control are essential to rapidly and efficiently respond to external stimuli without losing balance. Specifically, head–trunk separation is a sensorimotor development and visual anchorage-related skill and, consequently, upper body coordinative impairments are frequently detected in this target population [9,20,24,25,26]. Hip–trunk–head coordination allows the effective transfer of forces generated by lower body muscles along the total body kinetic chain underlying the main human motor patterns and balance control, which contributes to reducing joint overuse syndromes and injury risk [27,28,29]. Recent studies have highlighted the fact that trunk muscle strength and core stability/recruitment are associated with static and dynamic balance, daily living functional performance, and orientation skills in almost every age group, especially in visually impaired subjects [21,30,31,32]. Despite a remarkable lack of research specifically investigating this target population, it has been demonstrated that superior postural stability, gait efficiency, environmental mastery, and everyday functionality are directly related to higher levels of physical activity in those individuals [20,33,34,35]. Since visual input unavailability adversely affects kinesthesis abilities with consequent reduction in motor activity involvement and expertise of sight-impaired subjects [36,37], an intentional compensation/reeducation of these skills through targeted sensorimotor training protocols and regular sport practice are needed [21,38].

In this context, due to the performance model underlying the fundamental motor gestures and the multimodal input prevised by blind baseball (BXC) game dynamics and rules, such an adapted sport can significantly contribute to sensorimotor efficiency and quality of life improvement of regular practitioners [39,40]. As already detailed in our previous studies, the kinetic chain at the base of batting, running, and pitching requires finely orchestrated movements, muscle strength/power, proprioceptive postural control, global and segmental coordination, and sensory reactivity [21]. This blind-adapted discipline, conceived in Italy in the early 1990s, kept the dynamic features of the original sport played by sighted subjects, though introducing specific adaptations aimed to foster the safety and autonomy of disabled players during their athletic performance [41]. In particular, BXC players are driven by auditory and somatosensory input while performing all the above-mentioned technical gestures that involve complex cross-coordination skills and executive speed and, therefore, core recruitment and head–trunk separation become crucial for multisensory orientation and athletic efficiency during the game [21]. Batting and pitching gestures generally require a complex neuromuscular coordination and an effective sensorimotor timing/control to transfer ground reaction forces from the lower to upper body, thus conveying to the ball the maximal energy amount [42]. Within the kinetic chain on which these technical fundamentals are based, the swing movement represents the key ring, both for overload prevention and for athletic performance [43,44]. In sighted players, swing is mostly driven by visual input related to ball velocity or opponent batter running speed during the offensive and defensive phases of the game, respectively [45,46]. Conversely, in sight-impaired athletes, it primarily relies on proprioceptive information and involves upper body coordination/isolation patterns not naturally managed by those subjects. When consciously trained and acquired through a multimodal adapted approach, these motor skills can be easily transferred to daily life activities requiring multiplanar movements in the interaction with the tridimensional surrounding environment [21,39], hence counteracting disability-related deficits. Regarding BXC athletic performance, such prerequisites and motor abilities must be specifically trained during the whole sport season and especially boosted in the pre-season phase in order to prevent injuries and promote sport-specific body awareness and control [28,47,48].

On this basis, and taking advantage of the peculiar on-field expertise of our research team, the present study aimed to provide training methodological hints to improve the upper body sensorimotor control of visually impaired individuals through BXC anatomofunctional prerequisites and innovative workout tools. Among the latter, our investigation exploited a biofeedback-based proprioceptive tool (Libra sensorized board; Easytech, Borgo San Lorenzo, Florence, Italy) purposely designed to quantitatively assess postural control efficiency. The multisensory outputs provided by the digital interface allow the use of such an innovative device even in sight-impaired individuals, simply setting an auditory feedback instead of a visual one [21]. In addition, functional motor tests that are widely administered in healthy athletes were applied to reinforce the double aim, both re-educative and performative, of our proposal. Such a multiperspective tailored approach may help in boosting daily living functionality while contemporarily promoting sport practice and adherence in a non-medicalized enjoyable context in this vulnerable and under-investigated target population. In the wake of our previous studies addressing postural and motor control in visually impaired subjects [20,21], we expected that the proposed tailored training protocol could effectively improve the overall sensorimotor skills and the related anatomofunctional parameters in the investigated sample.

## 2. Materials and Methods

### 2.1. Study Participants

The study participants included 8 visually impaired baseball players (mean ± SD age, 25.4 ± 9.1 years), 5 (62.5%) male and 3 (37.5%) female, from the Fiorentina BXC team regularly registered to Polisportiva Silvano Dani. In detail, 6 (75.0%) subjects were congenitally visually impaired while 2 (25.0%) had acquired vision loss. According to the Italian visual disability classification [49], blind and severely sight-impaired levels were the most equally represented categories (37.5%), followed by mildly sight-impaired (25.0%). Athletes were in possession of a valid sport medical certificate issued by a sports doctor, as mandatorily required by the Official Federation (FIBS), to take part in the regular competitive championship [41]. As commonly provided for Italian sport associations, in the act of renewing the annual membership to the team, each athlete signed informed consent and agreed to participate in the training and evaluation proposals promoted by the team management during the whole sport season. In such a perspective, with president’s approval, the training and evaluation protocol was conceived, supervised, and performed by the official technical staff of the team, specifically including the official adapted physical activity kinesiologist [21]. Since the sample consisted of a professional sport team whose athletes are regularly trained and measured at various stages of their competitive season, no formal approval by a properly constituted ethics committee was applicable. In agreement with the informed consent provided by all participants, the data were treated, processed, and stored in a completely anonymous form for the purposes of this study. The study was also performed according to the Declaration of Helsinki [50].

### 2.2. Participant Evaluations

Evaluation procedures were conducted before and after the ending of the adapted sensorimotor training (AST) protocol during the pre-season phase of the 2023 BXC regular championship, mostly applying validated quantitative tools, both traditional and innovative, to assess upper and lower body anatomofunctional prerequisites and global/segmental postural stability in the whole sample of visually impaired athletes.

**Figure 1:**
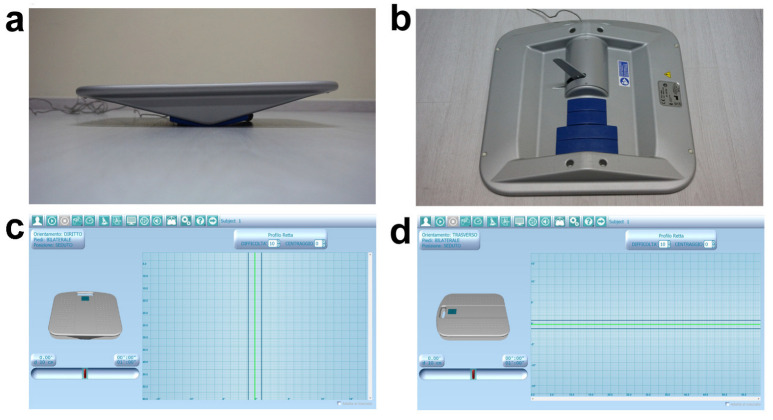
Libra sensorized proprioceptive board. (**a**) Adjustable tilting radius. (**b**) Sensorized arm and interchangeable tilting wedges. (**c**,**d**) Digital interface and board settings during lateral (**c**) and antero-posterior (**d**) trunk stability test.

All test batteries were performed strictly following the official guidelines available in the literature. Concerning the upper body, active range of motion (AROM) of the head was measured through the digital goniometer Easy Angle (Meloq AB, Stockholm, Sweden) in flexion, extension, bilateral rotation, and inclination movements [51]. Bilateral upper limb AROM was also assessed while performing flexion, extension, adduction, abduction, and internal and external rotation movements. In addition, trunk AROM was evaluated during bilateral twist around the longitudinal axis, first placing the subject in a sitting and then in a half-kneeling position [51]. It has been demonstrated that such a digital goniometer meets the highest clinical standards and has an excellent reliability and validity compared to the widely used manual tools, allowing us to obtain fast, consistent, and accurate joint AROM data [52]. For the purpose of trunk functionality assessment, three out of the seven movement tasks prevised by the Functional Movement Screen (FMS™) test were used [53]. In particular, trunk extensor/abdominal/lateral muscle endurance tests were performed and estimated by measuring, in seconds, the skill of maintaining the specific isometric position without any postural compensation. Recently, it has been demonstrated that this widely applied screening test may be considered a valid and reliable tool for global and district functional assessment of athletes [54,55]. Furthermore, upper body sensorimotor stability was investigated and quantified through the Libra sensorized proprioceptive board, an innovative biofeedback-based tool previously described and applied in our recent studies [21,56]. In the context of the present investigation, Libra was leant on a wood jump box and the subject was asked to sit over it, keeping their arms crossed over their chest, with a 90° trunk–thigh angle and both legs orthogonal to the ground with feet on a skimmy proprioceptive cushion placed on the floor. Starting from this body attitude, two tests were performed, the first with the device straight-oriented and the second one transverse-oriented, thus allowing the board to tilt on frontal and sagittal plane, respectively. Setting the aforementioned board orientation allowed us to investigate trunk lateral and antero-posterior stability. During both 1 min tests, a linear pathway pattern, a maximum 10/10 difficulty level, and 10 cm tilting wedges were set (Figure 1), while the aim was to keep Libra in balance, parallel to the floor, following the auditory feedback provided by the software. In order to obtain reliable values, the validated performance index prevised by the manufacturer was recorded at the end of each test. Specifically, it is calculated by the digital interface through the weighted average of eight values and must be interpreted referring to the validated 0–100 preset cut-off; lower values correspond to a better sensorimotor control. 

Regarding lower body district, the Thomas test was preliminarily performed to detect eventual lower limb anterior muscle chain retractions [57,58]; of note, no muscular deficits were observed in the whole sample. Additionally, bilateral hip goniometric AROM during internal and external rotation movements [51] were measured, as well as posterior muscle chain flexibility, through the well-known and validated sit-and-reach test [59]. Since orthostatic postural control plays a key role in daily living and sport functionality, the Libra Spielman-De Gunsch (SDG) test and the Y Balance test were also administered. This latter allows us to easily evaluate lower limb stability and functional symmetry through monopodalic multidirectional balance tasks performed following the three branches of a Y pattern drawn on the floor using a paper tape [60,61]. The subject, barefoot and placed in orthostatic position on the pivotal point of the Y with hands firmly on hips, was asked to keep monopodalic balance on the stance leg while sliding the controlateral foot as far as possible, subsequently following the different lines/directions of the drawn pattern. The required movement task must be executed by each lower limb following this mandatory direction order: right anterior, right posteromedial, left posteromedial, right posterolateral, and left posterolateral. Each monopodalic task was repeated for a total of three correct attempts; at the end of the whole test, the average achieved distance was recorded for each lower limb [60,61]. Since the sample consisted of visually impaired individuals, verbal explanation of the tasks followed by tactile exploration of the pathway were provided before performing the test. Finally, the SDG validated test was administered using the Libra sensorized proprioceptive board (Figure 1a,b) [62]. Such quantitative assessment has been purposely conceived to evaluate postural stability with respect to three different visual conditions, namely no gaze constraints, fixed gaze, and closed eyes. In case of sight-impaired subjects, the test can still be used simply applying head coordinative constraints (i.e., no head position constraint, straight head position constraint, and closed eyes). In detail, the SDG test previses three tasks of 30 s each with 25 s of recovery and repositioning in between, presetting a linear pathway pattern, a 9/10 difficulty level, and 10 cm tilting wedges. The subject, in orthostatic bipodalic stance, was asked to keep the board in balance, parallel to the floor, following the auditory feedback provided by the software. At the end of the whole test, the stability index obtained in each trial can be compared to the 0–100 cut-off values available in the digital database. Notably, lower scores correspond to higher postural stability.

### 2.3. Adapted Sensorimotor Training

**Figure 2:**
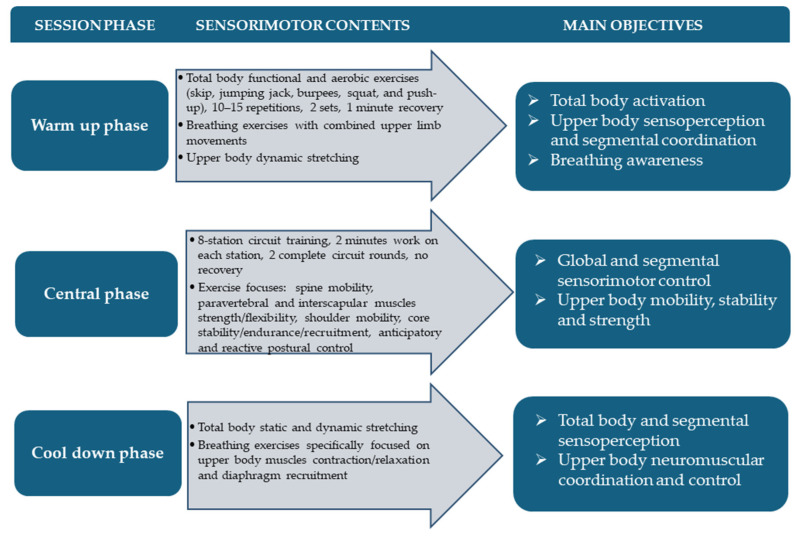
Adapted sensorimotor training organization.

The tailored training intervention was carried out on the whole Fiorentina BXC team during the pre-season stage of a regular sport season. Specifically, it was designed as two indoor 60 min workout sessions per week scheduled on non-consecutive days for a total protocol length of six weeks, from February to March 2023. The main objectives of our AST concerned trunk–head–pelvis coordination/separation, trunk stability and flexibility as well as global and segmental proprioceptive postural control. In order to improve these anatomofunctional prerequisites and sensorimotor skills contemporarily, respecting subjective fitness level and visual disability-related needs, a circuit training methodology was applied. As already reported in our previous studies addressing this target population, such an execution time-based methodological approach allows us to safely and individually adjust training load and to optimize communication and leading while taking advantage from the socio-emotional and motivational benefits of collective workout [20,21]. In detail, each training session was organized in three main phases, namely total body warm up, sensorimotor circuit training, and cool down. The first phase comprised aerobic activation through functional exercises such as skips, jumping jacks, burpees, squats, and push-ups along with breathing exercises with combined upper limb movements and upper body-focused dynamic stretching. The central phase consisted of an 8-station circuit training involving 2 min of work on each station for a total of two complete circuit rounds with no recovery between them. Particularly, exercises were focused on spine mobility on different anatomical planes, paravertebral and interscapular muscle strength/flexibility improvement, shoulder active mobility, anticipatory and reactive postural control, and core stability/endurance/recruitment. The third and final phase of each session was dedicated to total body static and dynamic stretching and breathing awareness, specifically focused on upper body muscle contraction/relaxation and diaphragm recruitment. Workout load was progressively increased by varying exercise executive positions, starting from unloaded ones until reaching BXC-specific body attitudes, introducing coordinative constraints and unstable surfaces (i.e., proprioceptive board, skimmy and hedgehog balance cushions, foam pad and fitball) and using small fitness tools such as sticks, elastic bands, ankle/wrist weights, light kettlebells, and dumbbells. In order to clearly summarize the training protocol design, an example workout session organization is graphically shown in Figure 2.

### 2.4. Statistical Analysis

All data are represented as mean ± standard deviation (SD), or number/percentage of subjects. The Wilcoxon signed-rank test was used to compare the baseline vs. post-adapted sensorimotor training (AST) intervention scores after verifying the normality of data with a Shapiro–Wilk test. The effect size of the comparisons (*r*) with 95% confidence interval was also determined. Values of *p* < 0.05 were considered statistically significant. Statistical analyses were performed using the SPSS version 29.0 (Statistical Package for the Social Sciences, Chicago, IL, USA). Statistical power was calculated with G*Power software (Version 3.1.9.7, Düsseldorf, Germany; online at http://www.psychologie.hhu.de/arbeitsgruppen/allgemeine-psychologie-und-arbeitspsychologie/gpower; accessed on 10 July 2024) [63]. The combined set of baseline and post-AST intervention variables provided a power of >80% for all comparisons.

## 3. Results

Results concerning the anatomofunctional assessment of upper body parameters at baseline and after ending the structured AST protocol are reported in Table 1. In detail, AROM values of head showed a post-intervention statistically significant improvement in flexion and extension, as well as in bilateral inclination and rotation movements (Table 1). Similarly, significant improvement in bilateral upper limb AROM was observed (Table 1). In addition, trunk AROM during right/left sitting and half-kneeling twist was significantly increased following the specific AST program (Table 1). Likewise, the trunk stability index, assessed by the sensorized Libra board on both the frontal and sagittal planes, resulted in significantly improved values (Table 1). Finally, core stability and recruitment, evaluated by the extensor/abdominal/lateral muscle isometric strength test, showed a statistically significant increase in all average scores (Table 1).

Table 2 shows the results of anatomofunctional assessment of lower body parameters at baseline and post-AST protocol. In particular, the posterior muscle chain flexibility, measured by the sit-and-reach test, was significantly increased following the specific AST program (Table 2). Moreover, bilateral hip AROM values showed also a statistically significant improvement at post-intervention respect to baseline in both external and internal rotation movements (Table 2).

Table 3 presents the data of postural stability, assessed either by Libra SDG or Y Balance test to provide an accurate assessment of the postural control quality, before and after the AST program in the study group. A statistically significant increase in all the postural stability test scores was observed (Table 3). 

## 4. Discussion

Research investigating sensorimotor control has often been focused on lower limb stability, given that the human body can be compared to an inverted pendulum with the ankles acting like a fulcrum [1,64]. However, since postural control efficiency is based on multisensory integration of the input provided by different perceptual channels, mainly located in the upper body [65,66,67], understanding the contribution of this anatomical district to such complex skills might be crucial to improving daily living functionality [68,69]. Despite the fact that it is well-known that vision significantly contributes to control/stability of trunk and upper extremities, existing literature on this topic in visually impaired individuals is still scarce [70]. 

To the best of our knowledge, this is the first study investigating upper body sensorimotor control in this target population through validated quantitative tools. By integrating BXC technical prerequisites within the herein-described AST intervention, we aimed to boost everyday functionality and autonomy, contemporarily preventing sport injuries and psychophysical overload [56]. The statistically significant results obtained in all the investigated parameters highlighted the preventive, re-educative, and training benefits of a tailored sensorimotor protocol on upper body functionality in physically active sight-impaired subjects. In particular, post-intervention trunk mobility/stability enhancement, detected through goniometric AROM and Libra proprioceptive board, might be strongly linked to core muscle recruitment and posterior muscle chain flexibility improvement observed in the FMS tasks and sit-and-reach test, respectively. The core includes trunk and pelvis muscles responsible for spine stability maintenance and force generation/transfer from large to small body districts during all human movements [71,72]. Furthermore, it connects upper and lower extremities via the abdominal fascial system, hence assuming not only a stabilizing but also a mobilizing function [73,74,75]. For such reasons, our AST protocol expressly focused on core recruitment and strengthening, also combined with upper and lower limb motion and unstable surface utilization [76,77,78], since it represents the fulcrum of the whole-body functional kinetic chain. Literature has repeatedly evidenced the importance of the core in optimizing force control and minimizing upper and lower body injuries, especially in overhead athletes [79,80]. Therefore, applying not only conventional but also sport-specific positions during the proposed workout tasks allowed subjects to easily transfer the acquired sensorimotor skills to BXC practice, thus preventing musculoskeletal overuse onset and improving the overall athletic performance. 

The purposely designed exercises focused on upper body sensoperception and reactivity to external perturbations, as well as breathing awareness [81], might have effectively contributed to achieving our post-intervention positive findings regarding trunk stability and mobility [21,56,82]. It has been demonstrated that balance performance and trunk neuromuscular reactivity in sitting position may be considered reliable core stability and global functionality indicators [72,73,83]. In this perspective, such complex skills have been investigated by applying sensorimotor sitting tasks performed on the Libra proprioceptive board, which allowed us to test them in a multisensory and dynamic real context [62]. The post-intervention improvement in trunk sitting twist AROM and Libra performance index, on both the frontal and sagittal planes, highlighted the effectiveness of multimodal and unstable/tilting tools in boosting upper body sensorimotor control and core recruitment in visually impaired individuals [20,84,85]. Specifically, the biofeedback-based technology offered by the Libra digital interface provides high frequency real-time feedback by transducing specific functional parameters into multisensory signals. Such technology allows us to consciously perceive and progressively control the micromovements of the whole body or its segments, hence improving anticipatory/reactive sensorimotor control and motor learning processes [56]. Sensorized proprioceptive devices such as the Libra board also allow us to quantitatively evaluate the subjective motor skills, thus facilitating the workout load customization [86]. Moreover, introducing specific coordinative constraints and promoting proprioceptive postural control during complex and dynamic motor tasks can effectively counteract disability-related head–trunk–pelvis coordination deficits and upper body stiffness [21,87,88,89], as demonstrated by the statistically significant post-protocol SDG test outcomes and head, upper limb, and hip mobility values. Since hip AROM highly contributes to body load management during orthostatic motor tasks [90,91,92], the observed improvement may be also related to dynamic balance improvement detected at post-intervention through the Y Balance test. Regarding this evaluation tool, it is noteworthy that our AST effectively increased not only global postural control but also lower limb functional symmetry, consistently reducing right and left difference between the average distance achieved performing it [61]. 

The main limitations of the present investigation can be identified mainly in the small size of the sample and the shortness of the proposed AST protocol. Nonetheless, it is important to remark that adapted physical activity interventions for visually impaired subjects must be led in small groups in order to safeguard the disability-related needs and grant safety in this target population [20,21]. Moreover, given the BXC-specific frame in which the study was carried out, it is important to remark that workout duration was set according to the pre-season phase length of a regular championship. Even though they refer to a small sample, all these encouraging short-term obtained results may represent a promising innovative approach to effectively testing and training this vulnerable demographic of individuals.

## 5. Conclusions

In conclusion, our findings suggest that a tailored AST intervention designed and leaded by a kinesiologist may effectively improve upper body anatomofunctional prerequisites and proprioceptive postural control in sight-impaired subjects. We are confident that the present study could help spreading research interest and easily reproducible methodological hints to be applied to this under-investigated population [35] in order to improve global and segmental functionality. Moreover, combining and integrating re-educative and sport-inspired perspectives could help in counteracting the disability-related multidimensional deficits while fostering regular physical engagement in a non-medicalized enjoyable context [20]. Indeed, the evaluative procedures detailed in the present study highlight the growing necessity of both adapting validated tools and designing innovative ones to objectively and safely investigate visually impaired individuals, hence not limiting research to clinical and rehabilitation contexts [20,93].

Finally, given the growing athletic performance levels required of BXC players, as a future perspective, our study could help foster the necessity of applying an evidence-based workout programming. In fact, such a kinesiologist-led approach could effectively and safely adjust methodological and motor contents to the different sport season phases and each team’s peculiarities [21].

## Figures and Tables

**Table 1:** Anatomofunctional assessment of upper body parameters at baseline and post-AST protocol.

Variables	Baseline Mean ± SD	Post-AST Mean ± SD	*p*-Value *	95% CI Lower Upper	*r*
AROM head, degrees					
Flexion	37.65 ± 4.98	52.25 ± 5.06	0.01	10.10 19.20	14.97
Extension	43.36 ± 5.29	57.16 ± 3.53	0.01	9.30 18.70	13.95
Right inclination	30.07 ± 6.96	41.45 ± 6.39	0.01	7.05 16.60	11.55
Left inclination	32.36 ± 5.96	42.41 ± 6.08	0.01	7.00 13.05	10.25
Right rotation	46.67 ± 9.36	65.56 ± 7.33	0.01	9.80 28.70	18.75
Left rotation	50.83 ± 9.09	66.56 ± 5.79	0.01	8.35 23.70	15.77
AROM right upper limb, degrees					
Flexion	147.38 ± 23.22	172.08 ± 11.95	0.02	2.65 41.80	22.25
Extension	39.28 ± 9.39	49.21 ± 1.29	0.03	2.75 17.35	10.05
Abduction	137.61 ± 32.20	172.56 ± 16.63	0.02	16.65 56.85	36.72
Adduction	26.95 ± 8.49	45.81 ± 13.55	0.01	10.75 27.00	17.87
External rotation	76.26 ± 10.10	91.91 ± 7.48	0.02	4.70 26.45	14.72
Internal rotation	72.68 ± 11.64	98.18 ± 5.99	0.01	7.80 28.10	17.05
AROM left upper limb, degrees					
Flexion	147.53 ± 18.15	176.80 ± 9.94	0.02	8.00 48.80	30.12
Extension	40.92 ± 7.59	49.86 ± 0.69	0.03	3.40 15.40	8.27
Abduction	139.00 ± 31.77	172.15 ± 21.52	0.03	12.25 54.10	34.05
Adduction	28.98 ± 10.65	48.83 ± 13.98	0.01	10.75 29.95	19.90
External rotation	78.86 ± 11.89	93.73 ± 10.61	0.04	0.00 33.40	11.10
Internal rotation	72.32 ± 14.62	89.68 ± 5.29	0.01	4.95 31.60	17.87
AROM trunk, degrees					
Right sitting twist	28.47 ± 6.82	51.08 ± 8.80	0.01	14.70 30.90	23.37
Left sitting twist	30.92 ± 8.70	50.38 ± 6.46	0.01	9.10 30.60	19.27
Right half-kneeling twist	31.88 ± 10.05	55.72 ± 8.91	0.01	13.60 34.45	23.72
Left half-kneeling twist	35.28 ± 9.52	55.62 ± 7.27	0.01	11.50 31.40	19.97
Libra performance index					
Frontal plane trunk stability	23.26 ± 5.47	14.15 ± 2.78	0.01	−12.18 −5.61	−9.94
Sagittal plane trunk stability	17.92 ± 5.16	12.17 ± 3.75	0.01	−9.72 −3.30	−5.33
Trunk isometric strength, seconds					
Extensor muscles	36.50 ± 29.76	63.75 ± 32.64	0.01	12.05 41.50	25.75
Abdominal muscles	33.62 ±17.92	55.00 ± 22.44	0.01	13.00 32.00	21.50
Right lateral muscles	20.37 ± 12.50	39.87 ± 12.57	0.01	6.50 31.00	20.75
Left lateral muscles	25.87 ± 9.70	42.62 ± 12.30	0.01	8.00 28.50	14.25

Abbreviations: AROM, active range of motion; AST, adapted sensorimotor training; SD, standard deviation of the mean; CI, confidence interval. * Wilcoxon signed-rank test.

**Table 2:** Anatomofunctional assessment of lower body parameters at baseline and post-AST protocol.

Variables	Baseline Mean ± SD	Post-AST Mean ± SD	*p*-Value *	95% CI Lower Upper	*r*
Sit-and-reach test, centimeters	38.28 ± 10.75	54.50 ± 9.03	0.01	8.50 26.40	16.00
AROM hip, degrees					
Right external rotation	41.48 ± 2.43	54.76 ± 4.49	0.01	9.00 18.00	13.17
Right internal rotation	31.96 ± 2.67	42.13 ± 4.38	0.01	6.60 14.50	10.12
Left external rotation	41.42 ± 3.89	52.75 ± 2.87	0.01	7.45 15.35	10.87
Left internal rotation	31.88 ± 3.40	40.81 ± 5.83	0.01	3.45 14.40	9.20

Abbreviations: AROM, active range of motion; AST, adapted sensorimotor training; SD, standard deviation of the mean; CI, confidence interval. * Wilcoxon signed-rank test.

**Table 3:** Mean scores of postural stability assessment at baseline and post-AST protocol.

Variables	Baseline Mean ± SD	Post-AST Mean ± SD	*p*-Value *	95% CI Lower Upper	*r*
Libra SDG test, index					
No constraint	15.86 ± 1.20	13.88 ± 1.97	0.01	−3.11 −1.03	−1.74
Straight head	15.54 ± 1.92	12.54 ± 1.98	0.01	−4.93 −1.55	−3.11
Closed eyes	15.34 ± 1.68	11.94 ± 2.09	0.01	−5.97 −0.95	−3.39
Y Balance test, centimeters					
Right	70.00 ± 11.32	96.12 ± 8.28	0.01	12.50 42.50	27.25
Left	67.25 ± 10.09	95.87 ± 7.51	0.01	17.00 41.50	27.00

Abbreviations: AST, adapted sensorimotor training; SD, standard deviation of the mean; CI, confidence interval. * Wilcoxon signed-rank test.

## Data Availability

The original contributions presented in the study are included in the article; further inquiries can be directed to the corresponding author.
